# Supplementary material for: An anomaly detection scheme for data stream in cold chain logistics
Source: PLoS One. 2025 Mar 10;20(3):e0315322. doi: 10.1371/journal.pone.0315322 (PMC11892858; doi:10.1371/journal.pone.0315322)
Supplement: Attachment — Submitted filename: Response to Reviewers.docx [file pone.0315322.s005.docx]

**Response to Reviewers**

Reviewer #1: COMMENTS
1. The abstract should provide specific numerical results from the experiments, such as precision, recall, F1 score, and AUC, to substantiate the claims of performance improvement

Thank you for your suggestion. I have added specific values for the experimental results in the abstract, including precision, recall, F1 score, and AUC.

2. Provide more context on the challenges and limitations of current anomaly detection techniques in cold chain logistics, specifically detailing why existing methods are inadequate.

I have add the challenges and limitations of current anomaly detection techniques in cold chain logistics and summarized the shortcomings of the existing technology.

3. Expand the literature review to include recent advances and a more comprehensive analysis of existing algorithms used in cold chain logistics.

I have add the literature review in the paper include recent advances and a more comprehensive analysis of existing algorithms used in cold chain logistics.

4. Provide a clearer and more detailed explanation of the improved isolated forest algorithm and the modifications made (subsampling and cross factor).

I have provided a more detailed introduction to the idea of improving the algorithm and added three core pseudocodes.

5. Ensure that all mathematical notations and equations are clearly defined and explained, with real-world examples if possible.

I have revised all mathematical notations and equations.

6. Elaborate on the derivation and significance of the correlation coefficient and how it is used to detect anomalies.

I have added explanations and derivations.

7. Describe the dataset in more detail, including its origin, size, type of data, and how it represents cold chain logistics.

I have added the dataset information.

8. Include a comparative analysis with more baseline algorithms and provide statistical significance testing (e.g., t-tests, p-values).

I think the performance indicators includes the *P*,*R*,*F1* score,AUC and ROC in the paper can verify the correctness of the algorithm, and I have also studied many similar articles during this period, and their methods for verifying algorithm performance indicators are basically similar to mine.

9. add more visualizations, such as ROC curves or precision-recall curves, to illustrate the algorithm's performance across different thresholds.

I have added the ROC curves in the paper.

10. Discuss the implications of the results in the context of real-world cold chain logistics scenarios. Include potential limitations of the proposed method and areas for future research.

The experiments datasets in the paper are from real-world cold chain logistics scenarios.

I have added the potential limitations of the proposed method and areas for future research.

11. Summarize the key contributions and explicitly state the practical impact and potential applications of the findings.

I have added the key contributions and explicitly state the practical impact and potential applications of the findings.

12. Review and refine the language for clarity, coherence, and conciseness. Ensure that technical terms are used consistently throughout the paper.

I have review and refined the language and expression.

Reviewer #2: The paper is appropriately organized and presented.
The research method is effective in anomaly detection for data stream of Cold Chain Logistics. The experimental data is sufficiently detailed.
There are some Innovation in anomaly detection for data streams.

However, there some minor symtax problems in Lines 114,140, 154, 205, 236, 251, 471, and so on. I hope the paper could be properly revised and make it understood easily.

Thank you very much. I have correct the mistakes.
